# The bilingual brain turns a blind eye to negative statements in the second language

**DOI:** 10.3758/s13415-016-0411-x

**Published:** 2016-02-29

**Authors:** Rafał Jończyk, Bastien Boutonnet, Kamil Musiał, Katie Hoemann, Guillaume Thierry

**Affiliations:** Faculty of English, Adam Mickiewicz University, Poznań, Poland; Leiden Institute for Brain and Cognition, Leiden University, Leiden, The Netherlands; School of Psychology, Bangor University, Bangor, Wales UK; Department of Psychology, Northeastern University, Boston, MA USA

**Keywords:** Affect, L2, ERP, N400, LPC

## Abstract

Neurobilingualism research has failed to reveal significant language differences in the processing of affective content. However, the evidence to date derives mostly from studies in which affective stimuli are presented out of context, which is unnatural and fails to capture the complexity of everyday sentence-based communication. Here we investigated semantic integration of affectively salient stimuli in sentential context in the first- and second-language (L2) of late fluent Polish–English bilinguals living in the UK. The 19 participants indicated whether Polish and English sentences ending with a semantically and affectively congruent or incongruent adjective of controlled affective valence made sense while undergoing behavioral and electrophysiological recordings. We focused on the N400, a wave of event-related potentials known to index semantic integration. We expected N400 amplitude to index increased processing demands in L2 English comprehension and potential language–valence interactions to reveal differences in affective processing between languages. Contrary to our initial expectation, we found increased N400 for sentences in L1 Polish, possibly driven by greater affective salience of sentences in the native language. Critically, language interacted with affective valence, such that N400 amplitudes were reduced for English sentences ending in a negative fashion as compared to all other conditions. We interpreted this as a sign that bilinguals suppress L2 content embedded in naturalistic L2 sentences when it has negative valence, thus extending the findings of previous research on single words in clinical and linguistic research.

Affect permeates social interaction, and is a faithful companion of our perceptions of the world (Bromberek-Dyzman, 2014; Kopytko, 2004; Zajonc, 1984). It is often at the origin of our thoughts, it determines behavior, and it influences decision making. Language is naturally a key means of expression and perception of affective information. In today’s multilingual world (Grosjean, 1984, 2010), proficient communication in more than one language is not just an asset, it has become the norm. This has important bearing on the investigation of affective language processing because drawing a faithful picture of bilingual communicative encounters will require distinguishing between native and nonnative language. This, in turn, may lay the foundation for formal communicative strategies, for instance, in legal or pathological situations. Previous studies have shown that affective experience may be contingent upon the language—first or second—of bilinguals in which information is communicated (Pavlenko, 2012). For instance, clinical studies focusing on the expression of emotions have consistently reported that the L2 represents a kind of asylum for patients discussing highly emotional and/or traumatic experiences (e.g., Aragno & Schlachet, 1996; Burbridge, Larsen, & Barch, 2005; Dewaele & Costa, 2013; Javier, 1995; Schwanberg, 2010). In a similar vein, healthy individuals often report their first language to be the “language of the heart,” in which affect is expressed and perceived with greater intensity (Dewaele, 2006, 2010; Pavlenko, 2005, 2006). Recent behavioral and psychophysiological studies, however, have provided inconsistent evidence for differential affective experience between the first and second language (Altarriba & Basnight-Brown, 2011; Conrad, Recio, & Jacobs, 2011; Eilola, Havelka, & Sharma, 2007; Ferré, Sánchez-Casas, & Fraga, 2013; Jończyk, 2015; Opitz & Degner, 2012; Ponari et al., 2015; Sutton, Altarriba, Gianico, & Basnight-Brown, 2007; Wu & Thierry, 2012). In an event-related potential (ERP) study, for instance, Opitz and Degner (2012) reported more pronounced early posterior negativity (EPN) amplitudes for emotional compared to neutral words in both the languages of bilinguals. The only difference was observed in the onset of the effect, with the EPN occurring later in L2. The authors suggested that emotional words in L2 might be processed less automatically. In contrast, Wu and Thierry (2012) reported that Chinese–English bilinguals fail to access the translation equivalent in Chinese of negatively valenced English words, or at the very least, that lexico-semantic access to the native language is hindered by negative words presented in L2, whereas positive and neutral words leave native lexico-semantic access intact. This result stands in contrast to previous evidence of implicit and unconscious access to translation equivalents in the native language upon reading or hearing words in English (Thierry & Wu, 2007; Wu & Thierry, 2010) and was tentatively interpreted as blocking of access to potentially harmful information by the bilingual brain (Wu & Thierry, 2012).

To the best of our knowledge, the investigation of electrophysiological correlates of affective language processing in context has so far received no attention in bilingual research, with relatively few studies addressing this issue in monolingual research (Bayer, Sommer, & Schacht, 2010; De Pascalis, Arwari, D’Antuono, & Cacace, 2009; Holt, Lynn, & Kuperberg, 2009; Martín-Loeches et al., 2012; Moreno & Vázquez, 2011; Rohr & Abdel Rahman, 2015). Studying language-affect interactions in richer contextual embedding (i.e., within sentences) could bring us closer to the conditions in which affective language is experienced by bilingual individuals in everyday life and perhaps help resolve inconsistencies in the literature. Implementing natural sentence context in research of affective language will thus provide a more faithful picture of affective interactions, resolve potential ambiguities in the meaning of decontextualized affective stimuli, and possibly enhance affective response to these stimuli (Rohr & Abdel Rahman, 2015).

Here, we investigated the electrophysiological and behavioral correlates of contextual affective language processing in 19 late fluent Polish–English bilinguals living in the UK. While undergoing electrophysiological recording, participants read Polish and English sentences that ended in either a congruent or incongruent affective adjective and indicated upon reading the last word whether or not each sentence made sense. Our analyses focused on the N400, a negative-going wave whose latency ranges between 200–600 ms poststimulus onset, peaking at around 400 ms over centro-parietal regions of the scalp.^1^ The N400 is known to index semantic integration difficulty (i.e., the cognitive effort required to integrate the meaning of individual words within the context of a sentence; Brown & Hagoort, 1993; Kutas & Federmeier, 2000, 2011; Kutas & Hillyard, 1980; Kutas, Lindamood, & Hillyard, 1984). Previous electrophysiological studies revealed qualitative differences in the way bilinguals processed sentences in their two languages (Elston-Güttler & Friederici, 2007; FitzPatrick & Indefrey, 2014; Frenck-Mestre, 2002; Hahne, 2001; Martin et al., 2013; Moreno & Kutas, 2005; Thierry & Wu, 2007). For instance, Martin et al. (2013) demonstrated that late fluent Spanish–English bilinguals reading sentences in their L2 English did not show an N400 incongruity effect for unexpected articles preceding sentence-final target nouns. The researchers concluded that fluent L2 readers might not anticipate the semantic resolution of sentences in the same way as native readers. Note that the overall N400 amplitude reported in the study, however, was more enhanced for L2 rather than L1 sentences. In a different study, Moreno and Kutas (2005) found similar N400 incongruity effect elicited by anomalous sentences in the L1 and L2 of Spanish–English bilinguals. The onset of the effect, however, was significantly delayed in the less proficient language of Spanish-dominant and English-dominant Spanish–English bilinguals. In the same vein as Martin et al. (2013), the overall N400 amplitude was greater for sentences in L2 than in L1. These findings contribute to the growing body of literature suggesting that the N400 effect might be qualitatively different in the two languages of bilingual individuals, with factors such as language proficiency, or age of L2 acquisition modulating the N400 wave (e.g., Ardal, Donald, Meuter, Muldrew, & Luce, 1990; Weber-Fox & Neville, 1996).

Critically, the N400 amplitude was also shown to be modulated by affective valence (De Pascalis et al., 2009; Herbert, Junghofer, & Kissler, 2008; Herbert, Pauli, & Herbert, 2011; Holt et al., 2009; Moreno & Vázquez, 2011; Wu & Thierry, 2012). For instance, De Pascalis et al. (2009) found more enhanced N400 amplitudes to negative compared to positive and neutral words embedded in sentences in monolingual participants. This effect was further modulated as a factor of participants’ impulsivity, whereby high-impulsive individuals displayed more pronounced N400 amplitudes to negative sentences than did low-impulsive individuals. Note, however, that the study did not dissociate between the effects of affective valence and semantic congruity. Therefore, the reported N400 effect might have been driven by semantic integration difficulty. This limitation was addressed by Moreno and Vázquez (2011), who found increased N400 amplitudes to positive compared to negative target words embedded in highly constraining positively biased and negatively biased sentence frames, respectively. The authors interpreted this effect as reflecting easier semantic integration for negative rather than positive sentences.

To our knowledge, our study is the first to test for a modulation of electrophysiological responses by affective valence during semantic integration in bilinguals. Based on existing research, we hypothesized that N400 amplitudes might be increased and/or delayed for sentences read in English as compared to those read in Polish in Polish–English bilinguals (Ardal et al., 1990; Martin et al., 2013; Moreno & Kutas, 2005). Critically, if affective processing were to be influenced by the language of input, we would expect an interaction between affective valence and presentation language during semantic access. Given the high levels of semantic and affective anticipation afforded by affective contextual information, the effect might unfold in the initial, early stages of semantic processing.

## Method

### Participants

Twenty-one native English speakers and 19 Polish–English bilinguals gave informed consent to take part in the study that was approved by the ethics committee of Bangor University, Wales, UK. All participants were right-handed and reported normal or corrected-to-normal vision. The bilingual group consisted of late immersed bilinguals currently residing in the UK. They reported using both Polish and English on an everyday basis, in both formal and informal contexts. The global proficiency rating for the two languages was established on the basis of self-reported reading, writing, speaking, and listening skills. Language history for the bilingual population was collected using the Language History Questionnaire (LHQ) 2.0 (Li, Zhang, Tsai, & Puls, 2014). Table 1 presents participants’ sociobiographical and linguistic information.

**Table 1:** Sociobiographical and linguistic information about mono- and bilingual participants. Measures represent means. Measures provided in brackets depict the standard error of the mean (*SEM*)

Measure	Monolinguals (7 ♂; 14 ♀)	Bilinguals (9 ♂; 10 ♀)
Age (at testing)	20.26 (.64)	24.36 (1.3)
Right-handedness score^a^	4.48 (.11)	4.76 (.07)
L1 self-rated proficiency^b^	n/a	6.59 (.27)
L2 self-rated proficiency^b^	n/a	5.8 (.18)
Age of L2 acquisition	n/a	11.17 (1.09)
Age at arrival in the UK	n/a	13.2 (1.3)
Length of immersion	n/a	8.06 (.18)

^a^established on a 5-point Likert scale, where 1 = *exclusively left*, 5 = *exclusively right*

^b^global proficiency rating was measured with a 7-point Likert scale on the basis of reading, writing, speaking, and listening skills, where 1 = *very poor*, 7 = *native like*

### Stimuli

Thirty-five positive and 35 negative adjectives were embedded in a sentence-final position of 140 constraining sentence frames, of three types: positive (*n* = 35), negative (*n* = 35), and neutral (*n* = 70). Sentences were further divided into four categories: (a) positive sentences ending in a congruent or incongruent word, both from a semantic and affective point of view (e.g., *Their honeymoon in the gorgeous scenery of Paris was so****romantic****/*burnt*); (b) negative sentences ending in a congruent or incongruent word (e.g., *Gloria accidentally poured boiling water over herself and was****burnt****/*romantic*); (c) neutral sentences ending in a congruent positive or incongruent negative word (e.g., *Women find him interesting, because Harry is very****romantic****/*burnt*); and (d) neutral sentences ending in a congruent negative or incongruent positive word (e.g., *Jerry spent a whole day in the sun and now his skin is****burnt****/*romantic*). In total, there were 140 congruent and 140 incongruent sentences whose length ranged from 8 to 15 words (*M* = 10.97, *SEM* = .13).^2^ Prior to the experiment, 42 individuals rated the predictability of all target adjectives on a scale from 1 (*unpredictable*) to 7 (*certain*). Congruent adjectives were rated as highly predictable (positive sentences: *M* = 5.54, *SEM* = .068; negative sentences: *M* = 5.50, *SEM* = .076) and incongruent adjectives were rated as rather unpredictable (positive sentences: *M* = 1.67, *SEM* = .064; negative sentences: *M* = 1.62, *SEM* = .066). Critically, positive and negative sentence endings were equally predictable, *t*(69) < 1. Neutral sentences were used as fillers and were not controlled for affective valence.

The mean valence and arousal ratings for the English stimuli were obtained from Warriner, Kuperman, and Brysbaert (2013). Positive target words (*M* = 7.42, *SEM* = .10) and negative target words (*M* = 2.47, *SEM* = .10) differed significantly in affective valence, *F*(1, 68) = 1125.46, *p* < .001, but not in arousal, *F*(1, 68) = .04, *p* = .842. We also collected ratings of affective valence for Polish and English target adjectives from our participants in a postexperimental norming study (see Procedure section) to increase stimuli reliability. Finally, we checked for potential differences in lexical frequency (positive: *M* = 4.27, *SEM* = .74; negative: *M* = 4.18, *SEM* = .69) and word length (positive: *M* = 7.85, *SEM* = .27; negative: *M* = 7.94, *SEM* = .29) between conditions by means of a one-way ANOVA and found no significant differences, frequency: *F*(1, 69) = 2.874, *p* = .095; word length: *F*(1, 69) = .377, *p* = .541. Lexical frequency ratings for English stimuli were obtained from SUBTLEX-UK (van Heuven, Mandera, Keuleers, & Brysbaert, 2014). Since at the time of designing the study there was no equivalent lexical frequency corpus for Polish words, we assumed rough similarity in lexical frequency measures between languages.

### Procedure

Participants were seated 100 cm away from a CRT monitor in a dimly lit and quiet room. They were asked to read sentences and to decide whether or not each one made sense upon reading the final word by pressing buttons on a response box. Expected endings were indicated by pressing a button with the nondominant hand (left). After a short practice run in the presence of the experimenter, participants completed two blocks of trials in English (monolinguals), or two blocks in English and two in Polish (bilinguals), the language of the first block being counterbalanced between Polish participants. A native Polish and a native English researcher were present at all times, enabling a short exchange in the language of the forthcoming block after each pause. Each block consisted of 70 sentences (35 congruent and 35 incongruent), and the same sentence was never repeated in the same language throughout the experiment. Participants first read the initial part of the sentence (a short phrase) and pressed a button to trigger the delivery of the second part in which words were presented one at a time until the end (target adjective). Each word of the second part was displayed for 200 ms in the center of the screen with an interstimulus interval (ISI) of 500 ms. The target adjective remained on the screen until participant response, but no longer than 2,000 ms, and it was preceded by a randomized interstimulus interval ranging between 350 and 700 ms in steps of 50 ms.

After the experiment, participants were asked to provide ratings of affective valence (1 = *extremely negative*, 7 = *extremely positive*) for Polish and English target adjectives that appeared in the experiment. Upon completion, they were debriefed and compensated for their time with £12 or five course credits. Postexperimental affective valence ratings are presented in Table 2.

**Table 2:** Postexperimental valence ratings for target adjectives

				Target valence	
		*F*_(1, 136)_	*p* (η^2^)	Positive	Negative
Valence	Overall	2856.415	.000 (.95)	5.78 (.36)	1.99 (.45)
	Polish			5.78 (.37)	1.99 (.48)
	English			5.77 (.36)	2.00 (.44)
	Language	.000	.989 (.00)		
	Val. × Lang	.014	.905 (.00)		

### ERP recording

Electrophysiological data were continuously recorded in reference to electrode Cz at a rate of 1 kHz from 64Ag/AgCl electrodes placed according to the extended 10-20 convention. Two additional electrodes were attached above and below the right eye to monitor and record ocular activity (eye blinks, vertical and horizontal eye movements). Impedances were kept <5 kΩ. EEG signals were amplified with Neuroscan SynAmps2 amplifier unit (El Paso, TX) and filtered online with a band pass filter between 0.1 and 200 Hz. Preprocessing steps and analyses were performed using MATLAB (R2012b, The Mathworks, Inc.) and a combination of scripts and routines implemented in the EEGLAB (v.13.3.2; Delorme & Makeig, 2004) and ERPLAB (v.4.0.2.3; Lopez-Calderon & Luck, 2014) toolboxes. Each dataset was down-sampled to 500 Hz and filtered offline with a 30 Hz low pass noncausal IIR Butterworth digital filter. Epochs ranging from -100 to 1,000 ms after the onset of the target word were extracted from the continuous, filtered EEG recording and subjected to visual inspection. Epochs with excessive muscular artifacts were manually rejected. Subsequently, an independent component analysis (ICA) was performed to extract and dismiss remaining ocular and muscular artifacts, following guidelines by Jung et al. (2000). No more than four independent components (ICs) were removed per participant. Finally, all epochs with activity exceeding ±75 *μ*V at any electrode site were automatically removed using a peak-to-peak moving window. The mean number of ICs removed and accepted epochs is summarized in Table 3. Baseline correction was performed relative to prestimulus activity, and individual averages were digitally rereferenced to the average of all scalp electrodes (global average reference).

**Table 3:** Mean and standard error of the mean (*SEM*) measures for preprocessing steps

	Monolinguals		Bilinguals	
	Mean	*SEM*	Mean	*SEM*
ICs removed	2.36	.20	3.13	.22
Valid epochs per condition	30.52	.60	30.82	.42

### ERP data analysis

We focused on the analysis of three ERP components: P1 and N1, to check for early effects of word affective valence, and N400, previously reported to be modulated by congruity, language, and/or affective valence (Kutas & Federmeier, 2000, 2011; Martin et al., 2013; Moreno & Vázquez, 2011; Wu & Thierry, 2012). The ERP components were therefore analyzed in the following predicted time windows and topographies: The P1 was analyzed over six electrodes (O1, O2, PO3, PO4, PO7, PO8) between 100 and 130 ms after final word onset (Thierry, Athanasopoulos, Wiggett, Dering, & Kuipers, 2009). The N1 component was analyzed over four electrodes (PO7, PO8, PO9, PO10) between 180 and 230 ms (Martin, Dering, Thomas, & Thierry, 2009). The N400 was analyzed over 10 electrodes (CP1, CP2, CPZ, C1, C2, Cz, FC1, FC2, FCz, Fz) between 280 and 550 ms (Kutas & Federmeier, 2000; Moreno & Kutas, 2005). Following visual inspection of the ERPs a component resembling a late positive complex (LPC) (Citron, 2012; Kissler, Assadollahi, & Herbert, 2006; Schacht & Sommer, 2009) was also analyzed exploratorily in the 600–800 ms time window on the 10 electrodes selected for N400 analysis (CP1, CP2, CPZ, C1, C2, Cz, FC1, FC2, FCz, Fz).

Statistical analyses were conducted within each participant group by means of repeated-measures ANOVAs with mean amplitudes as dependent variables and congruity (congruent, incongruent), affective valence (positive, negative) and language (English, Polish)^3^ as independent variables. A Greenhouse-Geisser correction was applied where appropriate and *p* values obtained from post hoc pairwise comparisons were adjusted using the Bonferroni correction. The data from two monolingual participants were excluded from the analysis due to excessive alpha rhythm contamination.

### Behavioral data analysis

A 2 (congruent, incongruent) × 2 (positive, negative) × 2 (English, Polish) repeated-measures ANOVA was conducted on reaction times (RTs) and error rates, with congruity, valence, and language as within-subject independent variables. The *p* values obtained from post hoc pairwise comparisons were adjusted using the Bonferroni correction.

## Results

### Behavioral results

#### Monolingual group

The ANOVA revealed a main effect of congruity, *F*(1, 20) = 5.870, *MSE* = 9831.79, *p* = .025, η^2^ = .22, whereby RTs were shorter for congruent (*M* = 850.61 ms, *SEM* = 30.10) than incongruent (*M* = 910.49 ms, *SEM* = 36.68) sentences. There was also an interaction between congruity and valence, *F*(1, 20) = 18.431, *MSE* = 3,939.40, *p* = .000, η^2^ = .48. Post hoc pairwise comparisons showed that it took longer for participants to judge that a sentence was congruent when it was negative (*M* = 883.11 ms, *SEM* = 31.31) as compared to positive (*M* = 818.11 ms, *SEM* = 33.07; *p* = .004). The reverse was observed for incongruent sentences where participants took more time to evaluate positive (*M* = 935.07 ms, *SEM* = 38.45) than negative content (*M* = 885.91 ms, *SEM* = 35.86; *p* = .001).

Error rates were modulated by an interaction between congruity and valence, *F*(1, 20) = 8.832, *MSE* = 43.67, *p* = .008, η^2^ = .30, such that participants were less accurate in judging a negative sentence as congruent (*M* = 12.51 %, *SEM* = 2.29) than a positive one (*M* = 7.75 %, *SEM* = 1.16; *p* = .026). In the incongruent condition, by contrast, positive sentences (*M* = 13.87 %; *SEM* = 3.43) were identified with marginally worse accuracy compared to negative sentences (*M* = 10.06 %, *SEM* = 2.13, *p* = .057).

#### Bilingual group

There was a main effect of congruity, *F*(1, 18) = 41.331, *MSE* = 11710.68, *p* = .000, η^2^ = .69, showing that RTs were faster to congruent (*M* = 826.56 ms, *SEM* = 29.24) than incongruent (*M* = 939.42 ms, *SEM* = 33.50) endings. There was also a main effect of valence, *F*(1, 18) = 13.068, *MSE* = 4,232.41, *p* = .002, η^2^ = .42, such that positive sentences elicited faster RTs (*M* = 863.91 ms, *SEM* = 28.96) than negative ones (*M* = 902.06 ms, *SEM* = 32.62). Crucially, our analyses revealed a significant 3-way interaction between congruity, valence and language, *F*(1, 18) = 7.021, *MSE* = 1,887.01, *p* = .016, η^2^ = .28. Post hoc comparisons showed that RTs were longer for congruent negative sentences in English (*M* = 867.54 ms, *SEM* = 48.13) and Polish (*M* = 860.48 ms, *SEM* = 36.06) relative to positive sentences both in English (*M* = 786.56 ms, *SEM* = 39.16; *p* = .001) and Polish (*M* = 791.65 ms, *SEM* = 34.29; *p* = .001). In the incongruent condition, no difference was observed between conditions (*p*s > .05).

**Fig. 1:**
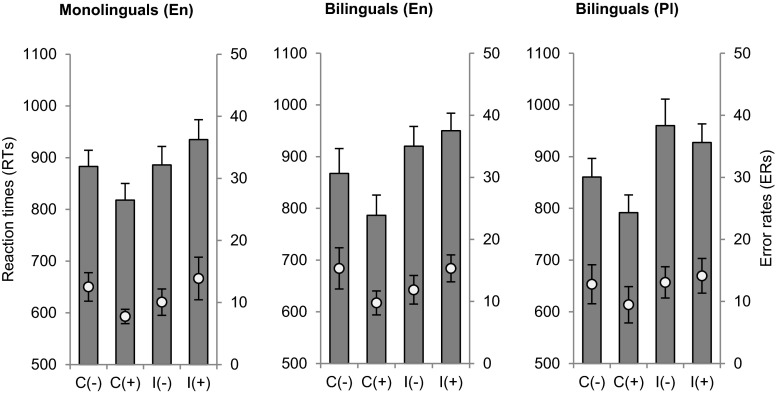
Mean reaction times (bars, left axis) and error rates (bullets, right axis) to negative (-) and positive (+) congruent (C) and incongruent (I) sentence targets in English monolinguals and Polish–English bilinguals. Error bars depict *SEM*

There was also an interaction between congruity and valence on error rates, *F*(1, 18) = 6.948, *MSE* = 61.23, *p* = .017, η^2^ = .27, such that participants made significantly more errors for congruent negative (*M* = 14.06 %, *SEM* = 2.57) than positive (*M* = 9.62 %, *SEM* = 2.02; *p* = .031) sentences. Figure 1 presents behavioral results for both participant groups.

### Electrophysiological results

#### Monolingual group

As expected, we found a significant main effect of congruity on the N400, *F*(1, 18) = 13.571, *MSE* = 1.06, *p* = .002, η^2^ = .43, such that amplitudes were increased for incongruent (*M* = .814, *SEM* = .47) relative to congruent (*M* = 1.688, *SEM* = .46) sentences. Valence did not modulate N400 amplitudes overall (*p* > .05). Valence and congruity interacted in the LPC range between 600 and 800 ms, *F*(1, 18) = 4.777, *MSE* = .95, *p* = .042, η^2^ = .21. Post hoc comparisons showed more pronounced LPC amplitudes to congruent negative (*M* = 3.038, *SEM* = .49) compared to incongruent negative (*M* = 2.329, *SEM* = .45) sentences. Other comparisons did not reach significance. Also, no significant P1 or N1 amplitude differences were found across conditions.

#### Bilingual group

**Fig. 2:**
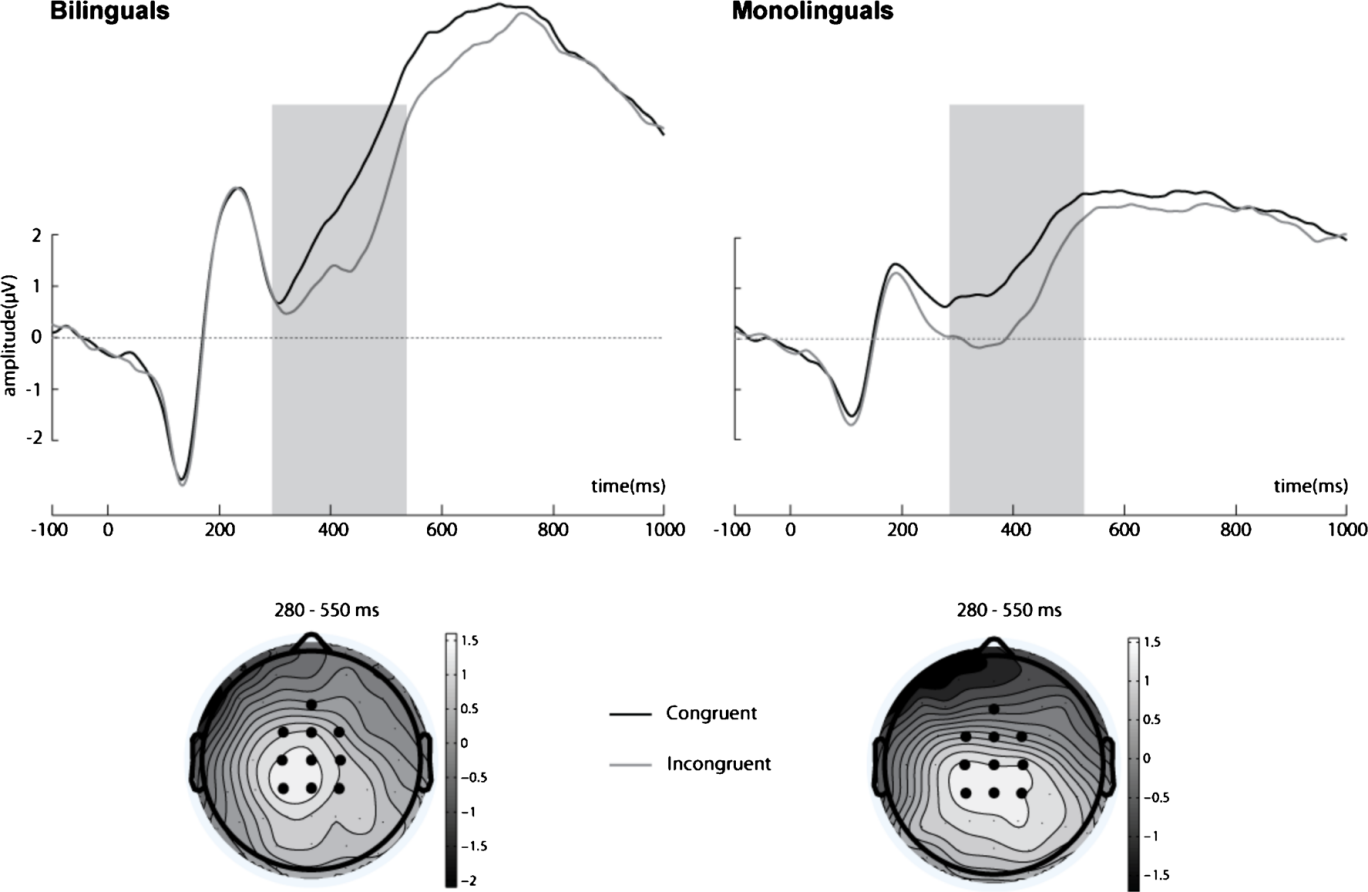
ERPs elicited by congruent and incongruent target words in Polish–English bilinguals and English monolinguals. All waveforms represent brain potential variations computed via linear derivation from 10 centro-frontal electrodes (CP1, CP2, CPZ, C1, C2, Cz, FC1, FC2, FCz, Fz). The shared gray area represents significant difference between conditions in the 280–500 ms time window. The schematic heads reflect difference topography of cortical responses to congruent minus incongruent target words 280–550 ms poststimulus onset at the electrode sites of interest

**Fig. 3:**
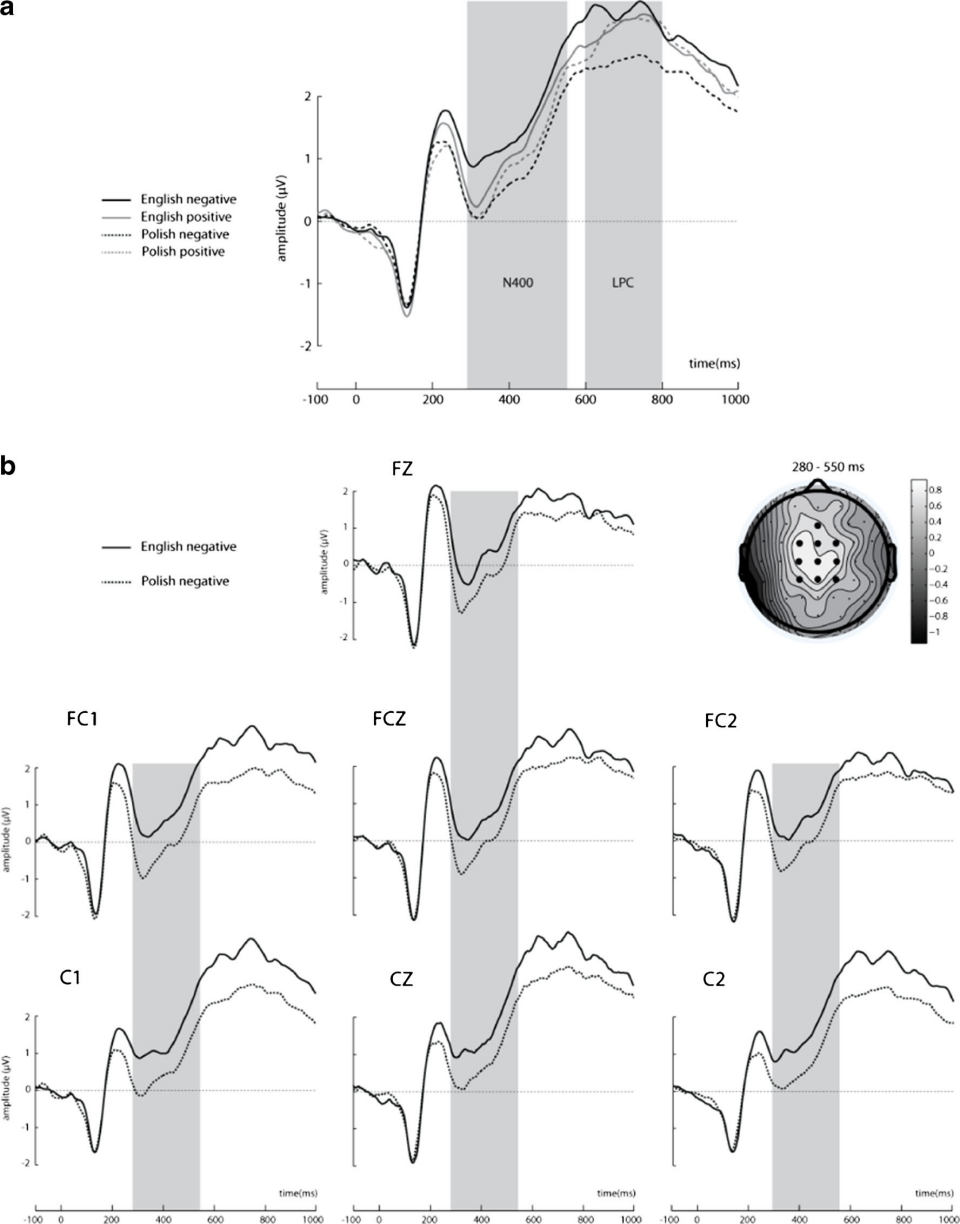

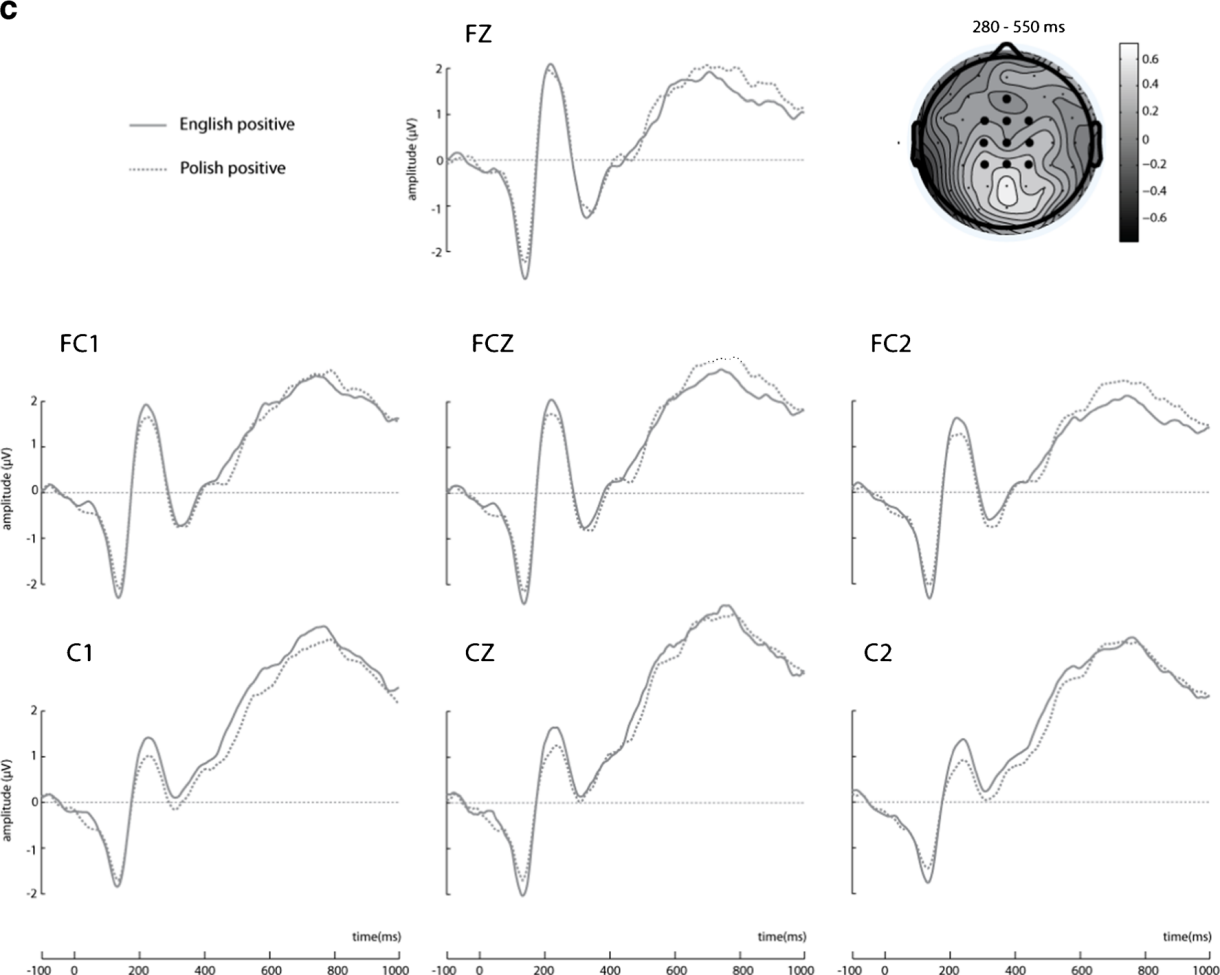
N400 and LPC elicited by target words in Polish–English bilinguals. A: Waveforms illustrate brain potential variations computed via linear derivation from 10 centro-frontal electrodes (CP1, CP2, CPZ, C1, C2, Cz, FC1, FC2, FCz, Fz). Shaded areas represent significant difference between conditions in the 280–550 and 600–800 time window. B: N400 at selected seven electrode sites where the effect was maximal. The schematic head reflects difference topography of cortical responses to English negative minus Polish negative target words 280–550 ms poststimulus onset at the electrode sites of interest. C: N400 at seven selected electrode sites where the effect was maximal. The schematic head reflects difference topography of cortical responses to English positive minus Polish positive target words 280–550 ms poststimulus onset at the electrode sites of interest

N400 amplitude was modulated by congruity, *F*(1, 18) = 15.849, *MSE* = .63, *p* = .001, η^2^ = .47; see Figure 2, with more negative amplitudes in response to incongruent (*M* = .847, *SEM* = .34) compared to congruent (*M* = 1.360, *SEM* = .30) sentences. Language of presentation also modulated the N400 amplitude, *F*(1, 18) = 5.843, *MSE* = 1.67, *p* = .026, η^2^ = .25, so that the N400 elicited by Polish sentences (*M* = .850, *SEM* = .33) was enhanced compared to English sentences (*M* = 1.357, *SEM* = .33). Critically, language and valence interacted in the N400 window, *F*(1, 18) = 7.403, *MSE* = .32, *p* = .014, η^2^ = .29; see Figure 3. Post hoc pairwise comparisons revealed that N400 amplitudes to negative sentences in English were significantly attenuated (*M* = 1.530, *SEM* = .35) compared to negative sentences in Polish (*M* = .773, *SEM* = .33; *p* = .002).

Furthermore, N400 amplitudes to negative English sentences were more attenuated compared to positive English sentences (*M* = 1.183, *SEM* = .32; *p* = .021). We found no other significant effect in this time window. Note that the language × valence interaction was unlikely to be artefactually driven by semantic incongruity, given the absence of a valence × congruity interaction (*p* = .870).

**Fig. 4:**
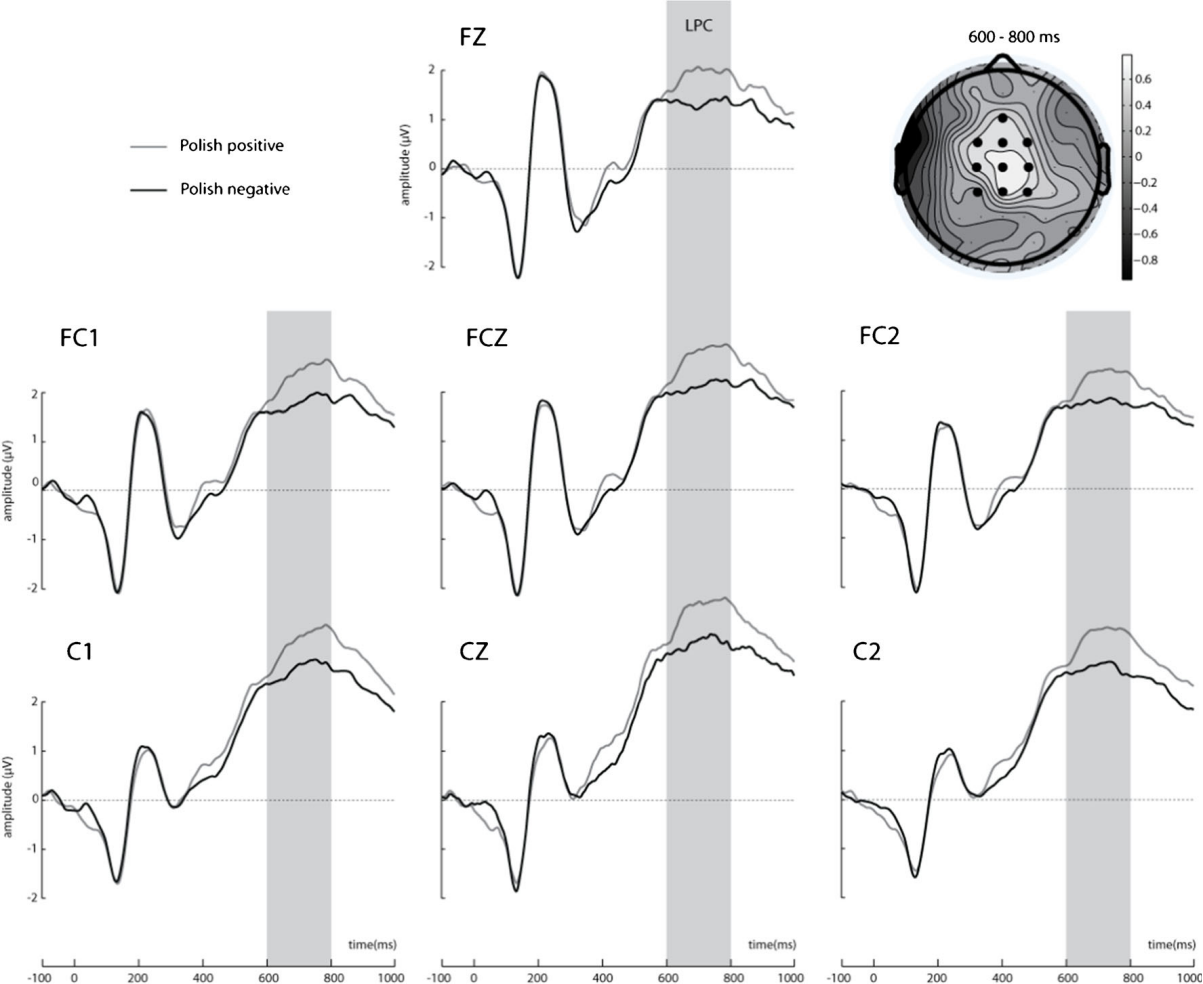
LPC at selected seven electrode sites where the effect was maximal. The schematic head reflects difference topography of cortical responses to Polish positive minus Polish negative target words 600–800 ms poststimulus onset at the electrode sites of interest

The language × valence interaction was also found in the analysis of the LPC, *F*(1, 18) = 8.007, *MSE* = .72, *p* = .011, η^2^ = .30; see Figure 4. Follow-up pairwise comparisons revealed that positive Polish sentences (*M* = 3.091, *SEM* = .42) elicited enhanced LPC amplitudes as compared to negative ones (*M* = 2.552, *SEM* = .40; *p* = .005). Also, we found more pronounced LPC amplitudes for negative English sentences (*M* = 3.363, *SEM* = .39) than negative Polish sentences (*p* = .024). No other effects were found in this time window. We found no significant P1 or N1 amplitude differences between any of the conditions. Waveforms illustrating responses to congruent and incongruent sentences at 30 electrode sites are available as supplementary material at https://osf.io/63etx/.

## Discussion

The present study investigated how Polish–English bilinguals immersed in a second language environment process the affective content of sentences. We expected to observe (a) greater semantic integration difficulty in the second language, as indexed by N400 amplitudes (Elston-Güttler & Friederici, 2007; FitzPatrick & Indefrey, 2014; Frenck-Mestre, 2002; Hahne, 2001; Martin et al., 2013; Moreno & Kutas, 2005; Thierry & Wu, 2007), and (b) differential N400 modulation by affective valence (e.g., Holt et al., 2009; Moreno & Vázquez, 2011; Wu & Thierry, 2012) in the first and the second language (given the use of naturalistic sentences more prone to evoking an affective response) as reflected by an interaction between valence and language.

Consistent with our critical hypothesis, we found a robust, differential N400 modulation by affective valence in L1 and L2 starting as early as 280 ms poststimulus onset, with negative English sentences eliciting reduced N400 amplitudes relative to (a) positive English sentences, (b) positive Polish sentences, and (c) negative Polish sentences, an effect independent of that of semantic congruity. Taking the view that the human brain anticipates both the semantic and the affective resolution of a sentence (Barrett & Bar, 2009; Martin et al., 2013; Martin, Garcia, Breton, Thierry, & Costa, 2014; Moreno & Vázquez, 2011; Van Berkum, Holleman, Nieuwland, Otten, & Murre, 2009; Wager et al., 2008), our finding suggests that semantic access to negative words presented in the second language of bilinguals may be incomplete and suppressed at an early stage of processing—that is, early in the course of semantic integration. This finding is consistent with the hypothetical mechanism of repression proposed for single words by Wu and Thierry (2012).

Using an implicit translation-priming paradigm, Wu and Thierry (2012) found that fluent Chinese–English bilinguals failed to display language unconscious nonselective lexical access from English to Chinese when the prime of a word pair presented in English had a negative valence, whereas the expected level of priming was found for word pairs primed by a positive or a neutral word. Consistent with Wu and Thierry’s (2012) results, also, we found no manifestation of the valence effect in the behavioral results.

Here, we extend the finding to semantic information presented in a sentence context and in a different language pair. We believe that the predictive context afforded by a more natural sentential context increased the demands on the semantic system and thus made it more likely to observe a direct affective modulation of semantic integration. However, we found an unexpected modulation of the N400 in the opposite direction that was expected. In addition, we observed a differential ERP modulation by affective valence in L1 and L2 also between 600 and 800 ms poststimulus onset (i.e., in the LPC range, known to index semantic reevaluation and reallocation of attention to affective stimuli; Citron, 2012; Kissler et al., 2006). Interestingly, the LPC showed the reverse pattern of that found for the N400, with negative sentences eliciting reduced LPC amplitude in L1 as compared to L2 and compared to positive sentences in L1. This might suggest that even though semantic access was unconstrained in the native language, reevaluation and reanalysis were differentially inhibited at a later stage. This mirror effect makes sense if one considers that affective information suppressed early on requires more processing at a reevaluation stage. Alternatively, it might be that, having fully appreciated the unpleasant nature of the negative sentence completion in L1, the brain fails to engage in reevaluation to the same extent as it does for positive and L2 words. Future studies are needed to validate this interpretation, however.

Our interpretation might be seen as one that departs from the way N400 effects have been typically interpreted in the literature (i.e., N400 amplitude indexing the difficulty of semantic integration; see Brown & Hagoort, 1993; Kutas & Federmeier 2011). However, based on classical findings from the L2 literature, one would have expected greater N400 amplitudes for L2 stimuli (Martin et al., 2013; Moreno & Kutas, 2005; Thierry & Wu, 2007). Thus we chose to interpret this effect as a sign of shallower processing in the case of L2, as has been shown for instance by Chwilla, Brown, and Hagoort (1995); Bentin, Kutas, and Hillyard (1993); or Hahne and Friederici (2002).

Together, our electrophysiological data offer new evidence consistent with the repression mechanism proposed by Wu and Thierry (2012), who used single emotional words in L2: the similar N400 amplitudes elicited by negative and positive sentence-final words in L1 suggest that semantic access is unconstrained for negative L1 words presented within a natural and affective sentence context, while the LPC results may suggest that the reevaluation and reanalysis is reduced for these same negative words, possibly indexing a late-stage protection mechanism after coping with earlier semantic access. By contrast, and in line with Wu and Thierry (2012), semantic access appears suppressed for L2 negative words and, thus, greater amplitudes in the LPC range because, having been processed more shallowly in earlier phases, these words would require more reevaluation.

We believe that these observations were made possible largely due to the implementation of an affective sentential context in our design that fosters anticipation and thus enhances interactions between affective and cognitive processing. Indeed, the human brain is highly predictive and constantly monitors situation development to act as fast as possible (Bar, 2007; Clark, 2013; Lupyan & Clark, 2015, Van Berkum, 2010). Consistent with this view, our results demonstrate that individuals not only predict how a sentence unfolds at a purely semantic level but also create specific expectations regarding its anticipated affective content (Moreno & Vázquez, 2011). We thus argue in favor of the implementation of a sentential context in the study of affective language processing because it likely enables subtle processing interactions between affective and semantic meaning that can hardly be uncovered by other means (Hinojosa, Carretié, Valcárcel, Méndez-Bértolo, & Pozo, 2009; Wu, Athanassiou, Dorjee, Roberts, & Thierry, 2012). Thus, in the present study, a negative sentence context would enable the anticipation of a negative final word with a potentially adverse effect, which would have reduced semantic access efficiency in the early stages of L2 processing and blocked the reevaluation of negative information in L1 at later, more explicit stages of processing.

Wu and Thierry (2012) speculated that a repression mechanism of lexico-semantic access may be underpinned by an interaction between the limbic system and the caudate nucleus, which is known to play a key role in bilingual language control (Abutalebi & Green, 2007; Ali, Green, Kherif, Devlin, & Price, 2010). Considering that our study used sentences, the richer contextual embedding likely leads to a greater involvement of the limbic system than in the case of single words presented out of context. Thus, if Wu and Thierry’s hypothesis is correct, we expect our more naturalistic paradigm to induce negative meaning suppression to a greater extent or sooner in the course of stimulus processing, leading to early modulations of the N400. Although the exact nature of the suppression mechanism for negative information in the second language is not yet established, it may provide a neurocognitive interpretational framework for findings from the clinical (Aragno & Schlachet, 1996; Burbridge et al., 2005; Dewaele & Costa, 2013) and introspective (Pavlenko, 2005, 2006) studies. Specifically, suppressed early access to negative information presented in the second language and subsequently inhibited reevaluation of negative meaning in the first language might explain why bilingual speakers find it easier to talk about traumatic events from the past in their nonnative language and/or subjectively describe their second language as emotionally detached. To date, however, most behavioral and psychophysiological experiments have failed to replicate findings reported in introspective studies (Eilola et al., 2007; Ferré et al., 2013; Jończyk, 2015; Ponari et al., 2015; Sutton et al., 2007), with essentially no measurable differences in affect processing between first and second languages. Through the implementation of natural sentential context in our paradigm, we were able to observe, for the first time, robust and dynamic differences in the processing of affective content between the L1 and L2 of Polish–English bilinguals.

Beyond the question of interactions between affect and language, we believe our results inform domains such as decision making, known to be under the influence of affect (Damasio, 2008; Gigerenzer, 2008). Recent studies by Keysar, Hayakawa, and An (2012) and Costa, Foucart, Arnon, Aparici, and Apesteguia (2014) showed for instance that bilingual speakers presented with a problem in their second language tend to be less affected by the consequences of a loss (i.e., display reduced loss aversion). In other words, they appear to be less affected by the negative consequences of a decision when reasoning is held in a nonnative language. Furthermore, Costa, Foucart, Arnon, et al. (2014) reported that bilinguals tend to be more objective and consistent when making decisions in their second language, probably due to greater emotional detachment. In two other studies, Costa, Foucart, Hayakawa, et al. (2014) and Geipel, Hadjichristidis, and Surian (2015) also reported that being in a nonnative language “mode” might differentially impact moral judgment. For instance, Costa, Foucart, Hayakawa, et al. (2014) reported that bilinguals make more utilitarian decisions in the second language (e.g., are more likely to sacrifice one individual to save five lives), possibly as a result of a greater affective detachment in L2. Geipel et al. (2015) demonstrated that individuals tended to be more lenient and at the same time less confident when making moral judgments in the nonnative relative to the native language. The authors attributed this effect to a possible inhibition of social and moral norms in L2, and only marginally so to the affective detachment in L2 (Geipel et al., 2015).

Along the same lines, Gao, Zika, Rogers, and Thierry (2015) tested whether risk-taking would be modulated by language-based feedback when participants repeatedly chose between playing or leaving (not playing) 50/50 gambles to win small monetary rewards. The choices were presented in numeric form but the outcome was presented using adjectives with positive and negative valence in the participants’ first language (Chinese) or second language (English). Gao et al. (2015) modeled the effects of presenting feedback indicating good and bad outcome in either Chinese or English upon participants’ subsequent decisions to play. Although positive feedback incited participants to play more on the next trial than negative feedback did, they found a striking dissociation between languages such that positive feedback in the native language, Chinese, incited participants to take 10 % more gambles (i.e., more risk) on the next trial as compared to all other conditions. This result converges with the results discussed above, in the sense that operation in a second language context reduces excessive emotional responses leading to increased risk taking in the native language.

In sum, substantial evidence showing deep-rooted interactions between language and nonverbal aspects of cognition is already available. This study extends this field of research to the domain of affective content by showing that bilingual individuals may process negative content more shallowly in L2 than L1.

However, unlike previous studies that reported increased N400 amplitudes for stimuli presented in L2 (Martin et al., 2013; Moreno & Kutas, 2005; Thierry & Wu, 2007), or delayed sentence processing in L2 (Frenck-Mestre, 2002), or difficulties anticipating sentence-final words in L2 (Martin et al., 2013), we found an unexpected modulation of semantic integration by language, with N400 amplitudes overall more negative in L1 than L2. This may be because affective content is generally more salient in the native language, leading to deeper processing indexed by greater N400 amplitude, which, in turn, would have canceled and even overcompensated differences in semantic integration difficulty between the first and second language. Note that, the magnitude of the N400 effect was similar in the Polish–English bilingual and English monolingual group, the only observable difference between the two groups in the N400 range relating to the onset of the N400 congruity effect (i.e., earlier in the monolingual group). This might be due to the possible differences in semantic anticipation in the first and second language, as already demonstrated by Martin et al. (2013).

Given the long history of immersion of our bilingual participants in the L2 environment (8 years on average) we predict that the reported effects would be more pronounced in participants who have spent less time in an English-speaking environment, because differences between Polish and English operating context would necessarily be more marked if their English was weaker and/or less contextually reinforced. Future studies may empirically validate this prediction. Ideally, future studies will also compare bilingual groups differing in their degree of L2 proficiency. Furthermore, in this study, neutral context sentences were used as fillers and their number was insufficient to allow meaningful statistical comparisons with affective conditions. Follow-up studies might thus involve investigating differences between affective and neutral sentence contexts and possible interactions with language of operation. Also, it would be interesting to test whether the effect of affective suppression is more robust when bilinguals listen to rather than read natural affective sentences.

## Conclusion

This study offers an opportunity to piece together evidence from clinical and introspective studies in bilingualism, emotion, behavioral, and psychophysiological research (see Pavlenko, 2012). Using affectively realistic sentence contexts enabled us to show that access to negative information in the second language may be suppressed in early phases of processing and triggers greater levels of reevaluation in later stages (which need not be effective). By contrast, early semantic access appears unconstrained in L1, and thus requires lesser levels of reevaluation at later stages. Such findings are likely to have significant implications for therapy, education, and everyday life in an increasing multilingual world.

## Footnotes

1 We also initially hypothesized that the valence-by-language interaction might already be detectable in early stages of lexical access (i.e., within the N2 range; Costa, Strijkers, Martin, & Thierry, 2009; Hoshino & Thierry, 2011) and spread to the N400 time window. This prediction related to the high level of affective and semantic anticipation afforded by the sentential context in the present experiment. Data analysis did reveal the predicted early negativity, but its scalp topography and latency was essentially overlapped with the N400 effect on which the analysis thus focused.

2 The stimuli are available as supplementary material at https://osf.io/63etx/.

3 The monolingual group was tested in English only, and therefore the language differentiation did not apply here and in further analyses.
